# Association of Elevated Glycated Hemoglobin (HbA1c) in COVID-19 Patients Admitted to the Intensive Care Unit and Their Clinical Outcomes

**DOI:** 10.7759/cureus.39599

**Published:** 2023-05-28

**Authors:** Adan M Cuevas Velazquez, Wern Lynn Ng, Evelyn J Calderon Martinez

**Affiliations:** 1 Internal Medicine, University of Pittsburgh Medical Center, Harrisburg, USA

**Keywords:** outcome measure, diabetes mellitus, hyperglycemia, a1c, covid-19

## Abstract

Aim

The study aimed to collect retrospective data to investigate the association between elevated glycated hemoglobin (HbA1c) levels and clinical outcomes in COVID-19 patients admitted to the ICU, including in-hospital mortality and 90-day mortality.

Methods

This is an observational retrospective study using electronic health records of patients with diabetes admitted to the ICU with COVID-19 across the University of Pittsburgh Medical Center (UPMC) in Central PA Hospitals. Our retrospective analysis was performed on patients admitted to the ICU between May 1^st^, 2021, to May 1^st^, 2022. The HbA1c level obtained within three months before their admission was evaluated and stratified to show their association with clinical outcomes, including in-hospital mortality and 90-day mortality. Additionally, the need for insulin drip and ICU and hospital length of stay were compared among these patients.

Results

We analyzed 384 patients, which were distributed in three groups. The majority of the patients (183 patients or 47.66%) had an HbA1c below 7%, 113 patients (29.43%) had an HbA1c between 7-9%, and 88 patients (22.92%) had an HbA1c above 9%. The group with an HbA1c<7% had a mortality rate of 54.1% during the hospital stay, with a median stay of 13 days. The patients with an HbA1c between 7-9% had a higher mortality rate of 65.49% with a median stay of 12 days. The patients with HbA1c>9% had a mortality rate of 43.18% with a median stay of 11.5 days.

Conclusion

This retrospective study found that there was no linear association between higher HbA1c levels and a higher risk of mortality during hospitalization. The 90-day mortality rate was not statistically different among the three HbA1c groups. The need for insulin drip was higher in patients with higher HbA1c levels. The majority of patients in all three groups were classified as low-risk based on their BMI, and there were no significant differences in the distribution of patients across BMI categories in the HbA1c groups.

## Introduction

Poor glycemic control is common during critical illness and adds another twist to the highly complex care given to patients in the intensive care unit (ICU) setting. Uncontrolled hyperglycemia can indirectly determine the prognosis or disease severity in critically ill patients [1]. Homeostasis and euglycemia are normally achieved by a variety of hormonal pathways in non-critical patients. Critical illness like COVID-19 can lead to a disturbance in the hypothalamic-pituitary-adrenal axis, thus leading to hyperglycemia. Chronic hyperglycemia can be further exacerbated due to increased gluconeogenesis via neuroendocrine hormone release in the critically ill, in addition to vasopressors and exogenous corticosteroids. There is not enough information on how chronic hyperglycemia can impact clinical outcomes in critically ill patients with COVID-19 but individuals with diabetes are at a higher risk of severe COVID-19 illness and mortality [2,3]. SARS-CoV-2 can cause inflammation that affects insulin sensitivity. Both hyperglycemia and hypoglycemia can further weaken the immune system and increase the risk of complications and mortality [4]. The objective of this study was to collect retrospective data to investigate the association between elevated glycated hemoglobin (HbA1c) levels and clinical outcomes in COVID-19 patients admitted to the ICU, including in-hospital mortality and 90-day mortality. Additionally, we compared the ICU and hospital length of stay and the need for insulin drip. 

## Materials and methods

This is an observational retrospective study using electronic health records of patients with diabetes admitted to the ICU with COVID-19 across the University of Pittsburgh Medical Center (UPMC) in Central PA Hospitals. Our retrospective analysis was performed on patients admitted to the ICU between May 1st, 2021, to May 1st, 2022. The HbA1c level obtained within three months before their admission was evaluated and stratified to show their association with clinical outcomes, including in-hospital mortality and 90-day mortality. Additionally, the need for insulin drip and ICU and hospital length of stay were compared among these patients. 

Patients with diabetes mellitus ≥18 years of age admitted for COVID-19 requiring ICU level of care and who had HbA1c level obtained within three months before their admission were included in the study. All patients <18 years of age, with HbA1c level obtained >3 months prior to admission, with hemoglobin level <12, who stayed in ICU less than <24 hours, transferred from another acute care facility, and discharged to hospice care were excluded from the study. 

The primary outcomes were to investigate how poor glycemic control (measured as an elevated hemoglobin A1c) before admission is associated with worse clinical outcomes, including mortality and the impact on patients' ICU/hospital length of stay. The secondary outcomes were to investigate the impact of poor glycemic control in different groups based on factors like age, sex, comorbidities, BMI, and the need for insulin drip for glycemic control. 

We reported continuous variables as means and standard deviations (SD) or medians and categorical variables as proportions. We used Student's t-test to analyze between-group differences and ANOVA or Kruskal-Wallis test for three group comparisons, as appropriate. Chi-square tests or Fisher's exact test was used for categorical variables. A multiple logistic regression model was used for the primary outcome adjusted for predefined baseline covariates: age, gender, and HbA1c level. All analyses were performed with the use of SAS software, version 9.4 (SAS Institute, Cary, USA). A p-value ≤0.05 was considered to indicate statistical significance. 

## Results

We analyzed 384 patients, which were distributed in three groups. The majority of the patients (183 patients or 47.66%) had an HbA1c below 7%, 113 patients (29.43%) had an HbA1c between 7-9%, and 88 patients (22.92%) had an HbA1c above 9%. 

In the group with HbA1c<7%, 111 were males (60.66%), and 72 were females (39.34%) with a median age of 64.2 years. They had a mortality rate of 54.10% during their hospital stay, with a median stay of 13 days. Insulin drip was required by 20.77% of them. 

The patients with HbA1c between 7-9% had a higher mortality rate of 65.49%, with 73 males (64.60%) and 40 females (35.40%) having a median age of 64.6 years. They had a median stay of 12 days. Insulin drip was required by 56.64%. 

Patients with HbA1c>9% had a mortality rate of 43.18%, with 59 males (67.05%) and 29 females (32.95%) having a median age of 59.1 years. They had a shorter median stay of 11.5 days. Insulin drip was required by 72.73%. 

The difference in 90-day mortality in the three groups was not statistically significant (p=0.5291). The need for an insulin drip was higher in patients with higher HbA1c levels (p<0.0001). 

We also distributed the patients in three different groups based on their BMI categories: low risk (<35), moderate risk (35 - 39.9), and high risk (≥40). The groups were further divided based on their HbA1c levels. In the group with HbA1c<7%, 124 patients (67.76%) were classified as low risk based on their BMI, 24 patients (13.11%) were classified as moderate risk, and 35 patients (19.13%) were classified as high risk. In the group with HbA1c between 7-9%, 67 patients (59.29%) were classified as low risk, 23 patients (20.35%) were classified as moderate risk, and 23 patients (20.35%) were classified as high risk. In the group with HbA1c >9%, 57 patients (64.77%) were classified as low risk, 10 patients (11.36%) were classified as moderate risk, and 21 patients (23.86%) were classified as high risk. 

Overall, the majority of patients in all three groups were classified as low-risk based on their BMI. In the group with HbA1c>9%, there were more patients classified as high risk compared to the other two groups, but the differences in the distribution of patients across the BMI categories were not statistically significant (p=0.3056). 

The results of the analysis are presented in Table 1.

**Table 1:** Comparison by HbA1c status HbA1c - glycated hemoglobin; CKD - chronic kidney disease

	Group 1 (HbA1c<7%), n	%	Group 2 (HbA1c between 7 - 9%), n	%	Group 3 (HbA1c>9%)	%	p-value
Total number of patients	183		113		88		
Patient demographics							
Gender							0.5587
Female	72	39.34%	40	35.40%	29	32.95%	
Male	111	60.66%	73	64.60%	59	67.05%	
Age: mean (SD), range	64.2 (13.2)	27-92	64.6 (11.3)	35-91	59.1 (13.8)	20-89	0.0037
BMI							0.3056
Low risk (<35)	124	67.76%	67	59.29%	57	64.77%	
Moderate risk (35 -39.9)	24	13.11%	23	20.35%	10	11.36%	
High risk (≥40)	35	19.13%	23	20.35%	21	23.86%	
Use of insulin drip							
Insulin drip	38	20.77%	64	56.64%	64	72.73%	<0.0001
Mortality							
In-hospital mortality	99	54.10%	74	65.49%	38	43.18%	0.0066
Discharged alive	84	45.90%	39	34.51%	50	56.82%	
90-day mortality	2	2.38%	2	5.13%	3	6.00%	0.5291
Length of stay (LOS)							
Total LOS (days): mean, median	15.9	13	15.2	12	13.9	11.5	0.1529
ICU LOS (hours): mean, median	227.3	176	225.1	163	193.4	150	0.4345
Comorbid conditions							
Cancer	19	10.38%	14	12.39%	4	4.55%	0.1557
CKD	49	26.78%	28	24.78%	20	22.73%	0.7651
Liver disease	4	2.19%	2	1.77%	0	0.00%	0.4885
Lung disease	64	34.97%	41	36.28%	21	23.86%	0.1224
Heart disease	68	37.16%	48	42.48%	34	38.64%	0.6573
Immunosuppression	19	10.38%	10	8.85%	4	4.55%	0.2738

## Discussion

Three hundred and eighty-four patients with diabetes mellitus admitted to the intensive care unit with COVID-19 were studied to analyze the association of their glycemic control previous to their hospital admission (using their HbA1c level within the past three months prior to their admission). The distribution of patients in each group was similar regarding gender, age, and comorbidities.

Interestingly, there was no linear association between higher HbA1c levels and a higher risk of mortality during hospitalization. The difference in the 90-day mortality in the three groups was not statistically significant. The ICU and hospital length of stay were very similar across the three groups. Previous publications of COVID-19 patients showed that diabetes mellitus is a risk factor for hospital admission and that they are at increased risk of needing ICU level of care [5-7]. Our approach with this study was to further evaluate this population and try to identify a correlation with the degree of chronic hyperglycemia.

One of the limitations of our study is that our inclusion criteria restricted the size of our sample since we required a recent HbA1c level for diabetic patients admitted to the ICU with COVID-19. Further investigation by increasing the sample may be necessary to increase the statistical power and confirm these results. Since this is a retrospective study, there is always a risk of unrecognized bias or confounders. It may not be appropriate to generalize our findings to all individuals with diabetes and COVID-19, especially those with less severe forms of the illness.

These findings could lead to several interesting discussions and questions. For example, it is worth exploring why higher HbA1c levels did not lead to a higher risk of mortality during hospitalization. One possible explanation is that the clinical presentation of COVID-19 can range from mild to moderate to severe. Adjusting the groups based on the severity of illness could explain the lower mortality rate in the group with an HbA1c>9. It could also be that the patients with higher HbA1c levels were receiving more aggressive management of their diabetes, which could have led to better outcomes during their hospital stay. 

The need for an insulin drip was higher in patients with higher HbA1c levels with a clear linear correlation.

The patients were also distributed into three groups based on their BMI categories: low risk (<35), moderate risk (35 - 39.9), and high risk (≥40). The majority of patients in all three groups were classified as low-risk based on their BMI. In the group with HbA1c>9%, there were more patients classified as high risk compared to the other two groups, but the differences in the distribution of patients across the BMI categories were not statistically significant.

## Conclusions

This retrospective study of 384 patients with diabetes mellitus admitted to the intensive care unit found that there was no linear association between higher HbA1c levels and a higher risk of mortality during hospitalization. The 90-day mortality was not statistically different among the three HbA1c groups. The need for insulin drip was higher in patients with higher HbA1c levels. The majority of patients in all three groups were classified as low-risk based on their BMI, and there were no significant differences in the distribution of patients across BMI categories in the HbA1c groups. However, further investigation may be necessary to confirm these findings and explore other potential risk factors for mortality in this patient population.
